# Understanding the interactions between bacteria in the human gut through metabolic modeling

**DOI:** 10.1038/srep02532

**Published:** 2013-08-28

**Authors:** Saeed Shoaie, Fredrik Karlsson, Adil Mardinoglu, Intawat Nookaew, Sergio Bordel, Jens Nielsen

**Affiliations:** 1 Department of Chemical and Biological Engineering, Chalmers University of Technology, SE412 96 Gothenburg, Sweden

## Abstract

The human gut microbiome plays an influential role in maintaining human health, and it is a potential target for prevention and treatment of disease. Genome-scale metabolic models (GEMs) can provide an increased understanding of the mechanisms behind the effects of diet, the genotype-phenotype relationship and microbial robustness. Here we reconstructed GEMs for three key species, (*Bacteroides* *thetaiotamicron*, *Eubacterium* *rectale* and *Methanobrevibacter* *smithii*) as relevant representatives of three main phyla in the human gut (Bacteroidetes, Firmicutes and Euryarchaeota). We simulated the interactions between these three bacteria in different combinations of gut ecosystems and compared the predictions with the experimental results obtained from colonization of germ free mice. Furthermore, we used our GEMs for analyzing the contribution of each species to the overall metabolism of the gut microbiota based on transcriptome data and demonstrated that these models can be used as a scaffold for understanding bacterial interactions in the gut.

The gut microbiota functions as a metabolically active organ and digests dietary components that are indigestible for human cells which can then be absorbed and metabolized by the human body^1^. The gut microbiota is also involved in the stimulation of the immune system and in providing resistance to pathogens. Perturbation or diversion of the metabolic functions carried out in the gut microbiota can lead to the development of different disorders^2^. Metagenomic studies have shown that the gut microbiome is associated with human diseases such as obesity^3^, type 2 diabetes^4^,^5^ and atherosclerosis^6^, and the composition of the gut microbiota has been shown to be influenced by the diet, environment and age^7^.

Improvements in DNA sequencing technology and cost reductions open new possibilities to study the human microbiome in health and disease. 16S rRNA sequencing has indicated that the gut microbiota is mainly dominated by the phyla Bacteroidetes (17–60%) and Firmicutes (35–80%)^8^,^9^. Other key phyla in the human gut microbiota are Actinobacteria, Proteobacteria and Euryarchaeota^6^,^10^. Previous studies have shown that Firmicutes are increased and Bacteroides are decreased in obese mouse models^11^. Better understanding of the interactions between these phyla as well as with the host may provide valuable insights into the underlying mechanisms of the different disorders. These interactions can be mediated by the production of short chain fatty acids (SCFAs) (acetate, propionate and butyrate), hydrogen and methane which are of potential interests in our study.

The SCFAs absorbed through the gut epithelial cells, have strong effects on the energy regulation and the immune system of the host^12^. The relative absorption of SCFAs by the colon varies between 60–90%^13^,^14^, and oxidization of SCFAs can provide energy for colonic mucosa and may contribute up to 5–10% of the total energy in a healthy body^15^. Acetate is the main SCFA in the blood and plays a key metabolic role for peripheral tissues being a substrate for lipogenesis and cholesterol synthesis and propionate is the precursor preferred by the liver to regulate cholesterol synthesis and gluconeogenesis^12^. The other SCFA, butyrate is an important energy source for colonocytes and affects the human energy balance by influencing energy regulation^12^. Butyrate is used as a fuel metabolite by colonocytes, resulting in a high level of ATP production^16^. Additionally, it has been recently proven that butyrate has protective roles against colon carcinogenesis^17^. Different studies have shown that absorbed butyrate in the colonocyte, inhibits histone deacetylase which modulates the colorectal cancer cell lines growth by influencing apoptosis, cell proliferation and differentiation^17^,^18^,^19^,^20^.

The bacterial fermentation and the production of SCFAs in the human gut are strongly associated with the dietary pattern which is correlated with the composition of bacteria in the gut. Modulation of the gut microbiota composition and the diet have been characterized both in human and animal studies^21^,^22^. Gnotobiotic mice harboring ten sequenced bacteria have been used to study the effect of different diets on species abundance and gene expression. A statistical method was developed for prediction of the bacterial abundance and identification of important factors in each diet^23^.

In order to obtain an increased understanding of the complex interactions between diet, microbiota and the host phenotype, reconstruction of genome-scale metabolic models (GEMs) for representative species of abundant phyla in the gut microbiota can provide an integrative platform to bridge the gap between genotype and phenotype^24^. GEMs are collection of biochemical reactions that occur in an organism, reconstructed based on high throughput omics data and known biochemical reactions which provide a scaffold for the analysis of such data^25^,^26^. GEMs can be employed to evaluate factors that result in modulation of the gut microbiota metabolism, and eventually to design clinical interventions^27^.

Previously, the mutualistic interactions between two bacteria have been studied through stoichiometric constraint based modeling^28^. This study represented the first attempt to simulate the interplay between microbial communities by considering each metabolic network as a separate compartment. This was followed by a multilevel optimization framework for the metabolic modeling and analysis of microbial communities called OptCom^29^. These two approaches constitute a framework for simulating ecosystems, but these approaches have not been applied and adapted to model the human gut microbiota^30^.

Here, we used two recently available technologies including genome scale modeling and meta-omics for describing the metabolism in the gut ecosystem and its interactions with the host. Two well characterized bacteria, *Bacteroides thetaiotamicron* (*iBth1201*) and *Eubacterium rectale* (*iEre400*) as representatives of the two abundant phyla, Bacteroides and Firmicutes respectively, were chosen for GEM reconstruction. *Methanobrevibacter smithii* (*iMsi385*), as a methanogenic and dominant archaeon in the human gut microbiome was selected as a third species because it plays a key role in gut microbial metabolism of hydrogen^31^. Despite of low abundance of *M. smithii* in metagenomics studies, it has a significant role in the human gut by removal of hydrogen gas and production of methane. Removal of hydrogen gas is important to consider as it affects bacterial fermentation and energy harvesting^32^.

Each GEM was validated using experimentally observed data and used for simulations of a simplified gut ecosystem. During our simulations, we formalized mathematically two different scenarios and applied them for modeling of gut microbiota. In the first case, the composition of the diet and the species abundances in the microbiota are known and constitute the input of the model. We predicted the profile of compounds produced by the microbiota and hence represented metabolites that can be taken up by the host. This simulation is referred as the α-problem and a solution was found by minimizing the substrate uptake rate (Figure 1). In the second case, which is referred as the β-problem, we predicted the abundances of the different species in the microbiota as a function of the diet. Next, we employed our GEMs for the analysis of transcriptomics data that profile the gene expression data of *Bacteroides thetaiotamicron* and *Eubacterium rectale* responds to each other^33^ and identified the metabolic differences in each bacteria using Reporter Metabolites and Subnetworks^34^. Furthermore we identified the transcriptionally regulated reactions using a random sampling algorithm^35^. We provided a mechanistic interpretation to statistical findings provided by metagenomics through the integrating of gut ecosystem modeling, diet compositions, genomic and transcriptomic data (Figure 1). This leads to improved understanding of the relationships between diet, microbiota and host and hereby enables a rational design of prebiotic and probiotic treatments.

## Results

### GEMs reconstruction of three representative bacteria in the human gut microbiota

In order to reconstruct draft GEMs for *B. thetaiotaomicron*, *E. rectale* and *M. smithii*, we first identified species for which GEMs have already been manually reconstructed using a bottom-up approach. This led us to choose well-studied template GEMs, *Escherichia coli* (*i*AF1260), *Staphylococcus aureus* (*i*SB619) and *Methanosarcina acetivorans* (*i*VS941) as templates species for *B. thetaiotaomicron*, *E. rectale* and *M. smithi*, respectively^36^,^37^,^38^. Due to different phylogeny of the template models and selected species, intensive manual curation was applied on the draft models based on literature and databases such as KEGG^39^, BRENDA, and Seed models^40^ for the identification and correction of inconsistencies. Biologically defined metabolic tasks that occur in each bacteria were defined and successful simulation of each task was completed using the RAVEN toolbox^41^. These metabolic tasks include the production of amino acids, nucleotides and carbohydrate metabolism and are summarized in Supplementary Dataset 1.

**Figure 1:**
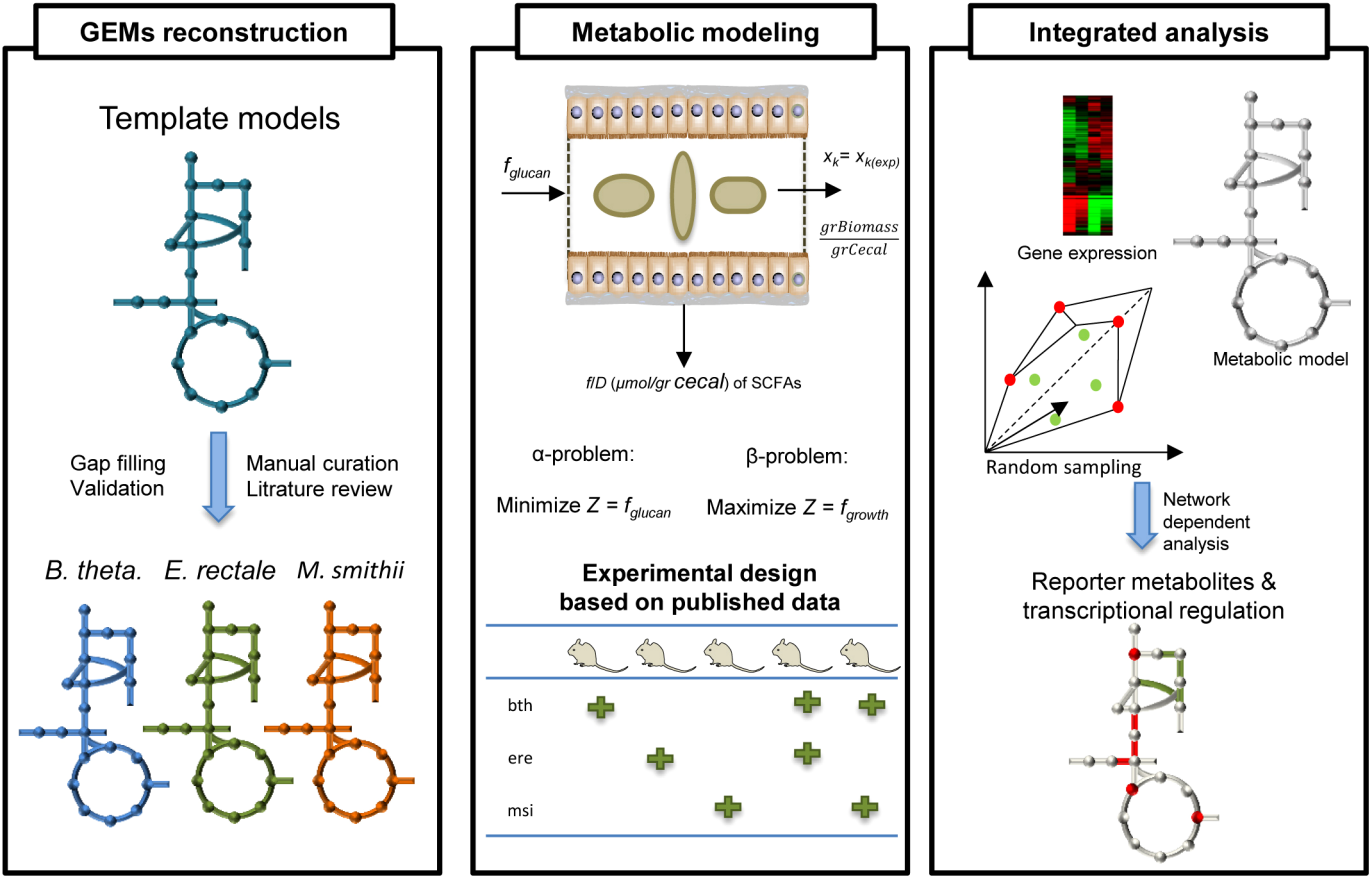
Genome-scale metabolic models (GEMs) for gut microbiota are reconstructed based on three template models and different databases. This was followed by intensive manual curation of the GEMs for three key species in the human gut microbiome, based on literature and databases. The metabolic modeling step involved two approaches, the α- and β-problem which were defined depending on the availability of experimental data (the mono-colonization of germ free mice with *B. thetaiotaomicron*^33^, *E. rectale*^33^ and *M. smithi*^47^, the co-colonization of germ free mice with the *B. thetaiotaomicron* and *E. rectale*^33^, the *B. thetaiotaomicron* and *M. smithii*^47^). In the α-problem the abundance of the species were specified and the glucan and the SCFAs uptake and secretion profile was predicted. For the β-problem, the concentration of substrates in the feed was specified and the biomass and the SCFAs profile were predicted. Finally, the metabolic differences between each bacteria were identified through integrative analysis. Reporter metabolites, subnetworks and random sampling algorithms were used to reveal information about transcription regulations based on the transcriptome data.

During the reconstruction process, we used genome sequences, previously published models and gene homology information as input (Figure 1). The homology information was obtained by performing a bidirectional BLASTp search between the organism of interest and the set of template organisms using standard settings. *iBth1201* was compared with recently published model for *B. thetaiotaomicron* *iAH991*^42^ and it is observed that *iBth1201* includes all of the genes and associated reactions in *iAH991* as well as 210 additional genes. The predictions obtained through *iBth1201* were in agreement with *in-vitro* chemostat data^43^ (Figure S1). The production of butyrate and the uptake of acetate and oligo and poly-saccharides were predicted through *iEre400* and were in agreement with *in-vitro* experiments^44^. Uptake of hydrogen, formate and acetate and production of methane were also predicted using *iMsi385*, which was also consistent with experimental observations^45^. The characteristics of each GEM are summarized in table S1 and these GEMs are publicly available through the Human Metabolic Atlas web-site (www.metabolicatlas.com).

### Solution of the α-problem in well-characterized gut ecosystems

The α-problem is defined as the determination of the secretion profile of metabolites as a function of the diet and the species abundances. Here, we solved the α-problem in a well-characterized gut ecosystem by integrating data on microbial composition from the literature and predicting the metabolites produced. A well-characterized gut ecosystem is defined as a microbial community in which the substrates and fermentation products of each bacteria are known. The microbial abundances (translated into gram biomass/gram luminal content) were inferred from humanized gnotobiotic mouse models. The α-problem was solved by fixing the biomass content of each organism to its experimental value and minimizing the substrate uptake. The interactions with the human gut were also considered by introducing experimental diffusion coefficients through the gut wall^46^. According to the available experimental designs, two different cases were simulated including single bacteria and the community of two microbial species. For both cases our simulation results were validated against experimental data^33^,^47^.

### *In-silico* evaluations of single species as a simple model for gut microbiome

**Figure 2:**
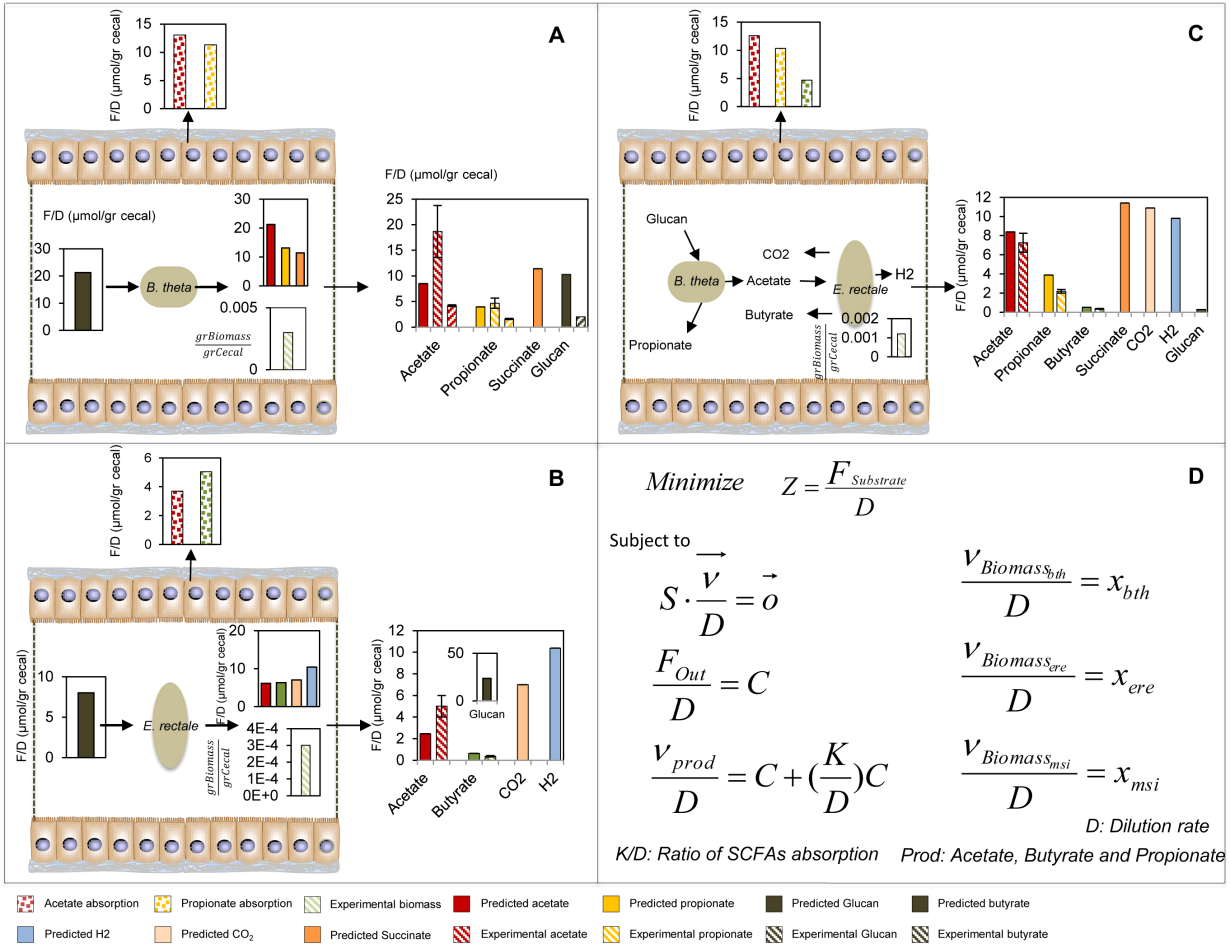
Comparison of the experimental measurements and predictions obtained from the solution of the α-problem for the ecosystem composed of *B. thetaiotaomicron* (A) *E. rectale* (B) and community of *B. thetaiotaomicron* and *E. rectale* (C) in colonized germ free mice. The green bar for different simulations indicates the abundant of species in the ecosystem. The total amount of available glucan for mice and produced acetate were calculated based on available experimental data. The amounts of absorbed SCFAs by the epithelial cells and the concentrations of SCFAs in the feces are shown. (D) The formulation for solving the α-problem is presented. The experimental data was reported in concentrations per unit of cecal content hence all the metabolic fluxes were divided by specific production rate of cecal content. The output fluxes are formulated in concentrations using the objective function. For all cases the initial glucan and acetate values are 31 and 6 μmol/gr cecal, respectively. The *x*_ere,msi,bth_ is the biomass experimental value for each species. (All units are F/D (μmol/gr cecal)).

**Figure 3:**
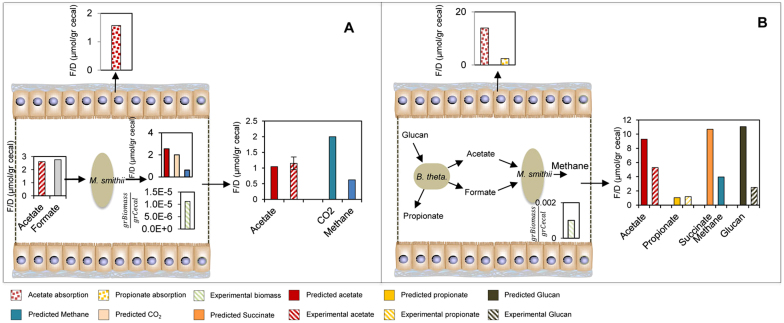
Comparison of the experimental measurements and predictions obtained from the solution of the α-problem for the ecosystem composed of *M. smithii* (A) and community of *B. thetaiotaomicron* and *M. smithii* (B) in colonized germ-free mice. The results are compared with predictions obtained from the solution of the α-problem. For community simulation the initial glucan and acetate values are 31 and 6 μmol/gr cecal, respectively. (All units are F/D (μmol/gr cecal)).

*iBth1201* was used to predict the profile of SCFAs and other byproducts and these predictions were compared with observed profiles of SCFAs in mono-colonized germ-free mice (Figure 2A). Predictions for two SCFAs (propionate and acetate) concentrations were in agreement with the experimental data. The deviation for acetate predictions and experimental values was 7.5 μmol/g Cecal and for propionate 1.5 μmol/g Cecal, which correspond to 25% and 22% deviation from the experimental value for acetate and propionate, respectively. Next *iEre400* was implemented as the sole gut bacterium and the concentration of butyrate, CO_2_ and H_2_ were predicted, and the results were compared with experimental data from colonization of germ-free mice with *E. rectale* (Figure 2B). The deviation between predicted and measured butyrate concentration was 0.07 μmol/g Cecal corresponding to 40% deviation from the measured concentration. Finally *iMsi385* was implemented to predict the consumption of acetate and production of methane by using constraint-based modeling (Figure 3A), and the simulations showed consistency with experimentally determined methane production.

### *In-silico* evaluations of two species ecosystems

Following evaluation of the individual models we simulated gut ecosystems involving two species. In the first case, when *E. rectale* is together with *B. thetaiotaomicron*, the main metabolite exchanged between the two organisms is acetate. In presence of *B. thetaiotaomicron*, *E. rectale* takes up small proportions of the acetate produced by *B. thetaiotaomicron* and convert it into butyrate. Very small proportions of carbohydrates are consumed by *E. rectale* and most are taken up by *B. thetaiotaomicron*. In order to model these interactions, the community stoichiometric matrix was built based on single stoichiometric matrixes. Here, the extracellular medium was treated as a common compartment between the two species. The same optimization formulation were implemented here (Figure 2D). The biomass concentration of each of the species in the community was fixed to its experimental value and constraint-based modeling was employed to minimize the substrate uptake. The SCFAs profile was compared to experimental values, where the deviation for acetate, propionate and butyrate were 0.7 (14%), 1.2 (40%) and 0.07 (30%) μmol/g Cecal, respectively (Figure 2C). In the second case the other combination of species, *B. thetaiotaomicron* and *M. smithii* was simulated. The main interactions between these two are the exchange of acetate and formate, which are taken up by *M. smithii* and ended up with production of methane by methanogenesis. Again in this case the biomass content of each community member was fixed to its experimental value. The secretion profile of the community was predicted as depicted in figure 3B by minimizing the substrate uptake rate and it was compared to the available experimental values. The deviation for acetate and propionate was 2.8 (40%) and 0.07 (14%) μmol/g Cecal respectively and these were within the range of experimental measurements (Figure 3B). To examine how the key predictions correlate with assigned biomass values, robustness analysis was applied for production of SCFA and consumption of glucan with different constraints of biomass. We defined a sensitivity coefficient (the ratio of biomass and SCFAs production changes for different constraints) which was close to one. This means that the relative error in the biomass estimation is equal to the relative error in the SCFAs prediction (Figure S2 & S3). Besides, the errors in the measured biomass concentrations are over 20%, which explains the relative errors in the prediction of short chain fatty acids. On the other hand (Figure 2A) the discrepancy in the experimental measurements of short chain fatty acids for different biological replicates (different mice) is also very high. Our predictions are as good as the quality and accuracy of the available experimental data allow.

### Solution to the β-problem for two and three species

The β-problem is defined as the prediction of both the biomass composition of the microbiota as well as the secretion profile of SCFAs with a known diet composition. The rational design of probiotics and prebiotics cannot be fully achieved without a methodology for solving the β-problem. Our *in-silico* method enabled testing the effects of different diets on the abundance of bacterial species present in the colon and their SCFAs secretion profile. The β-problem was solved for a community of *B. thetaiotaomicron* and *M. smithii*, by fixing the uptake rate for glucan to 20 μmol/g Cecal and maximizing the sum of biomass concentrations of *B. thetaiotaomicron* and *M. smithii*. The observed predicted values for the abundance of each species, SCFAs and methane production were in agreement with experimental data^33^,^47^. The abundance of *B. thetaiotaomicron* and *M. smithii* were 1.0 and 0.08 mg Biomass/g Cecal, respectively.

**Figure 4:**
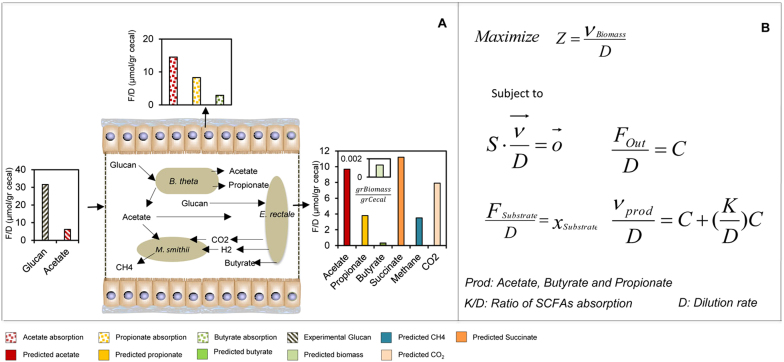
(A) Predictions obtained from the solution of β-problem for the ecosystem composed of *B. thetaiotaomicron*, *E. rectale* and *M. smithii*. In this case, the biomass content of the community used as an objective function. The profiles for SCFAs productions were obtained based on available amount of glucan. (B) The formulation to solve the β-problem (All units are F/D (μmol/gr cecal)).

To achieve further insight into the metabolic function of the human gut microbiota, the interactions of three species as representatives of predominant phyla were investigated. Solving the β-problem for three species leads to identification of the diet leading to the optimum SCFAs production which is in correlation with abundances of *B. thetaiotaomicron*, *E. rectale*, *M. smithii* (Figure 4A). The interrelationships of this simplified community were acetate production by *B. thetaiotaomicron* and consumption by *E. rectale* and *M. smithii* as well as CO_2_ production by *E. rectale* and consumption by *M. smithii*. The substrate was set and split between *E. rectale* and *B. thetaiotaomicron* which was observed from simulations with the α-problem and the objective function was defined as total growth of the microbial community (Figure 4B). By solving this problem corresponding SCFAs, abundances of each member of community and other byproducts such as methane and succinate were predicted. The patterns of SCFAs in this three species community were observed to be similar to the patterns in community with *E. rectale* and *B. thetaiotaomicron* (Figure 4A). This conceptual modeling result is the most realistic case in the human gut microbiota where SCFAs were produced, hydrogen gas was taken up and methane was produced by the *M. smithii*. The robustness analysis was also applied for the β-problem, where different values for glucan were assigned as an input to the community and the SCFAs and biomass of the community were predicted. The sensitivity coefficient (*S*) for this simulation was also found to be close to one (Figure S4 & S5).

### Integrative analysis of transcriptomic data for gut microbial communities

**Figure 5:**
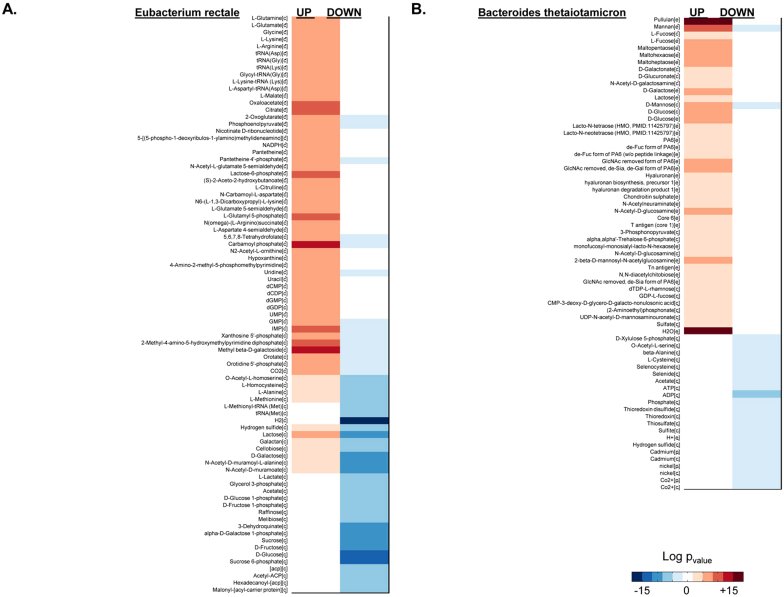
The reporter metabolites were identified for two conditions based on colonic transcriptional data^33^. (A) The response of *E. rectale* to *B. thetaiotaomicron* (B) response of *B. thetaiotaomicron* to *E. rectale*.

GEMs represent the link between genotype and phenotype and allow for generating testable hypothesis for cellular metabolism and for drawing novel biological conclusions based on omics data. In this context, we used a transcriptome dataset^33^ that profiled the transcriptome of *E. rectale* and *B. thetaiotaomicron* in monocolonized mice with a co-colonization of the two. We applied the reporter metabolites algorithm that identifies a set of metabolites around which there is observed a strong transcriptional response^34^. By generating the GEMs for these three species, using published gene expression data and applying network dependent analysis we used transcriptome data to get more insight at the mechanistic level (metabolite and gene level). We identified reporter metabolites, subnetworks and transcriptional regulation which have not been reported in the previous study. The use of GEMs and reporter algorithms is much easier for metabolic interpretation than manual looking through a list of genes. We presented reporter metabolites for *E. rectale* during its adaptation to *B. thetaiotaomicron* (Figure 5A) and reporter metabolites for *B. thetaiotaomicron* during its adaptation to *E. rectale* (Figure 5B). The reporter metabolites linked to the up and down regulated genes are presented. The analysis revealed that *E. rectale* responded to the presence of *B. thetaiotaomicron* by up-regulating the genes around the metabolites involved in amino acid metabolism, aminoacyl-tRNA biosynthesis, TCA cycle, NAD and CoA synthesis and nucleotide synthesis. These findings were is agreement with Mahowald *et al*^33^. We also observed that there is significant down regulation of enzymes in carbohydrate metabolism, such as melibiose, fructose, galactose and raffinose (Figure 5A & Supplementary Dataset 2). It should also be noted that the expression of the genes encoding enzymes interacting with L-alanine, L-methionine and L-homocysteine changed in both directions. *B. thetaiotaomicron* responded to the presence of *E. ractale* by up-regulating the expression of genes around the poly and mono-saccharides, carbohydrates and glycans ((Figure 5B & Supplementary Dataset 3). We also found that genes linked to beta-alanine, L-cysteine and selenide are down regulated.

**Figure 6:**
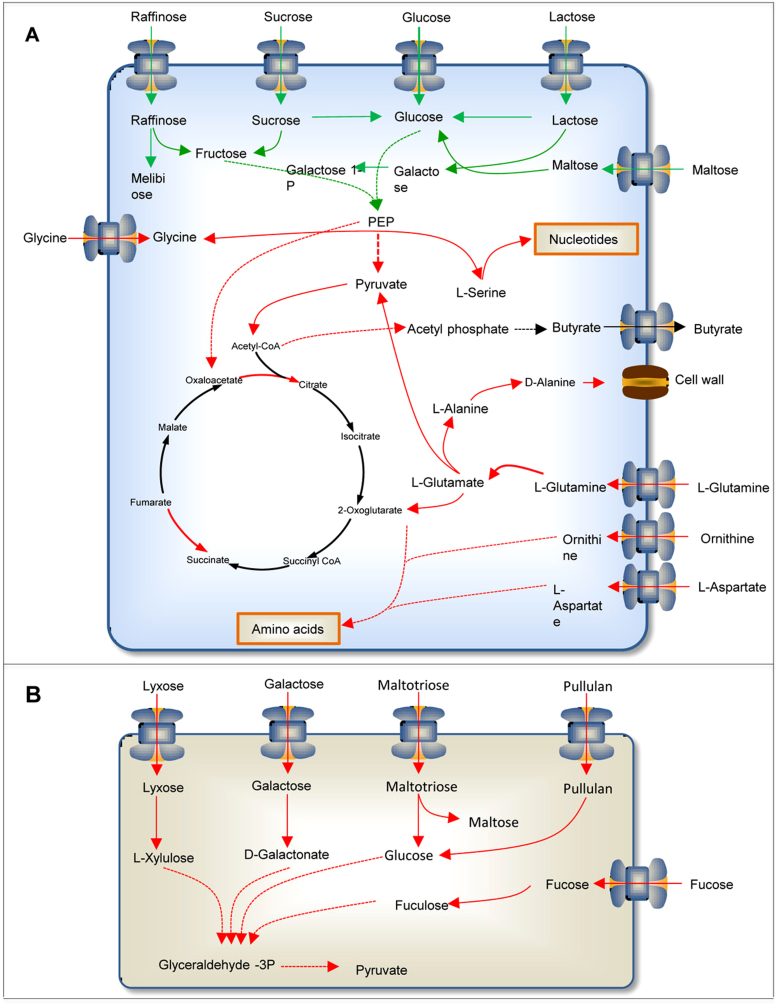
The reporter subnetworks for (A) *E. rectale* responds to *B. thetaiotaomicron* by increasing the utilization of amino acids and decreasing the degradation of carbohydrates (B) *B. thetaiotaomicron* responds to *E. rectale* by increasing the utilization of polysaccharides.

In order to gain more insights into the molecular mechanisms involved in the response of *B. thetaiotamicron* and *E. rectale*, the reporter sub-network algorithm was applied to identify set of metabolic reactions which exhibit transcriptional correlation. The identified reporter sub-networks are presented in figure 6 after removing highly connected metabolites (e.g. cofactors) (Supplementary Dataset 4 & 5). It is observed that *E. rectale* responds to *B. thetaiotaomicron* by increasing the utilization of amino acids and decreasing the degradation of carbohydrates (Figure 6A) whereas *B. thetaiotaomicron* responds to *E. rectale* by increasing the utilization of polysaccharides (Figure 6B). The complete reported subnetworks for both cases are presented in figure S6 &7.

### Revealing transcriptional regulation information

The regulatory mechanisms responsible for the changes in the distribution of metabolic fluxes between two states can be inferred using the GEMs by incorporating measured external fluxes and transcriptomic data in our random sampling algorithm^35^. Here, we used external fluxes predicted based on the α-problems and calculated a set of possible distributions of internal fluxes consistent with the secretion profiles for each of the considered conditions. We first evaluated the response of *E. rectale* to the presence of *B. thetaiotaomicron* and this analysis revealed that *E. rectale* transcriptionally up regulated the flux in amino acid metabolism, in particular reactions involving glutamine, serine, glycine, methionine, isoleucine, arginine and citrulline. Pyruvate decarboxylation, pyruvate phosphotransferase, 2-phospho-D-glycerate hydro-lyase and H2-oxidizing hydrogenase reactions were also identified as transcriptionally down -regulated (Supplementary Dataset 6). Reactions such as 2-aceto-2-hydroxybutanoate synthase are transcriptionally up-regulated, which reveals an adaptation to increased availability of acetate due to the presence of the other species. This shows the link between the high flux of butyrate production in presence of *B. thetaiotaomicron* and uptake of acetate by *E. rectale*. When *B. thetaiotaomicron* encounters *E. rectale* in the mouse gut, significant transcriptional up regulations of metabolism are seen mainly in glycolysis and the TCA cycle (Supplementary Dataset 7).

### Sensitivity analysis of *E. rectale* and *B. thetaiotaomicron*: Identifying optimal production of butyrate

The effect of different perturbations on the butyrate production which is a parameter relevant for the health of the host was identified through sensitivity analysis. This analysis was applied for *E. rectale* by varying the acetate and the glucan uptake rate and predicting the biomass and butyrate changes. In optimal condition, production of butyrate increases with higher uptake of acetate. The response of the butyrate production to changes in glucan and acetate is depicted in figures S8 and S9.

Another sensitivity analysis was applied on a community of *E. rectale* and *B. thetaiotaomicron*. In this analysis, the abundances of *B. thetaiotaomicron* and *E. rectale* in the community were varied to check optimal production of butyrate. Figure S10 shows how the butyrate production increases with the increased abundance of *E. rectale*. As mentioned above, butyrate is a key component that affects energy homeostasis and functions as histone deacetylase inhibitor, which has a direct effect on colorectal cancer. The results of this analysis can propose a solution for finding the threshold of butyrate formation based on the abundances of Bacteroides and Firmicutes.

## Discussion

In order to understand the function of bacteria in the human gut ecosystem and its interaction with the host, we reconstructed three GEMs, *iBth1201* (*B. thetaiotaomicron*), *iEre400* (*E. rectale*) and *iMsi385* (*M. smithii*), which are relevant representatives of three main phyla in the human gut (Bacteroidetes, Firmicutes and Euryarchaeota). We predicted the substrate uptake rates and the secreted SCFAs profiles for different combination of these three bacteria through *in silico* co-colonization of germ free mice. We simulated that the production of butyrate increased when *B. thetaiotaomicron* was co-colonized with *E. rectale*. This alteration of butyrate has different impacts on human health and it is involved in the progression of disorders such as colon cancer, diabetes and obesity^17^,^18^,^19^,^20^,^48^. In the joint simulation of *B. thetaiotaomicron* and *M. smithii*, the acetate produced by *B. thetaiotaomicron* and consumed by *M. smithii* as well as methane produced by *M. smithii* was predicted. It was observed that methane production was also increased in the community of *M. smithii* and *B. thetaiotaomicron* compared to *M. smithii* as the sole bacteria. The predictions of SCFAs production were compared to experimental data and they were in agreement with experimental observations.

In order to simulate the metabolic effect of the gut species on the host metabolism, we used experimentally measured diffusion coefficients for the absorption of each SCFA by the epithelial cells. It should be noted that the diffusion coefficients of SCFAs can differ based on individual genetic differences as well as the health status of the host. For all simulations, we used a fixed value for the diffusion coefficients (Acetate 60%, Propionate 70% and Butyrate 90%) based on previous studies^13^,^14^. We also demonstrated that it is possible to find the solution of the α-problem in well-characterized ecosystems where the exchange of metabolites by different bacteria is unknown a-priori. During our analysis, the exchange metabolites inferred from the structure of the metabolic models for the gut microbiota and the experimental biomass production of the species at minimal substrate consumption was simulated. The very good fit between our simulations and experimental data shows that the models correctly capture the electron balancing associated with the mixed acid fermentations occurring in the studied microorganisms, and hence provide validity to model simulations of more complex scenarios. This approach may therefore allow for inferring putative new interactions between the species forming the *in-silico* ecosystem of the human gut.

By integrating our bacterial community models with transcription data, we further demonstrated how we can identify transcriptional metabolic responses in each species for adaption to symbiotic conditions. By applying the two algorithms the mechanistic information was extracted from the transcriptome data compared with the study of Mahowald *et al*^33^. During the adaptation of *E. rectale* to presence of *B. thetaiotaomicron*, it was observed that the expression of *E. rectale*'s genes involved in the amino acid metabolism, TCA cycle and purine and pyrimidine metabolism were up regulated whereas genes involved in the degradation of carbohydrates are down regulated. Calculated Reporter Metabolite and subnetwork analysis supports this observation. During adaptation of *E. rectale* to presence of *B. thetaiotaomicron* it increased its dependency on the amino acid utilization, in particular glutamine that is the most abundant amino acid in the blood. Glutamine is a source of nitrogen and is used as a nitrogen donor for the biosynthesis of major biomass components e.g. nucleotides. Glutamine taken up by *E. rectale* is converted to glutamate and to other intermediates which are necessary for the synthesis of alanine, aspartate, arginine and proline. Previously the decreased blood level of glutamine in the PPAR-α null mouse which represents a number of discrepancies linked to diabetes and the metabolic syndrome^49^ and the significantly greater portion Firmicutes species are reported in obese mice^11^. Our results indicate that *E. rectale* in the gut may contribute to the decrease in the blood level of glutamine. The decreased glutamine level can be explained by the increased uptake of glutamine by Firmicutes species which is essential for increasing their growth.

During the adaptation of *B. thetaiotaomicron* to *E. rectale*, it increased its biomass depending on the polysaccharides. *B. thetaiotaomicron* yield energy for growth by fermenting non-digestible carbohydrates, e.g. cellulose, xylans, resistant starch and inulin in the colon and degrade these to SCFAs. The SCFAs produced from microbial fermentation can also be used as an energy substrate for host cellular metabolism.

Furthermore by mapping the flux distribution and expression data for two different conditions, significant enzymes with transcriptional regulation were identified which may further point to enrichment in activity of specific transcription factors. This type of analysis can be used to gain further insight into what drives the microbial ecosystem of the gut, and hereby assist with treatment of metabolic diseases that are associated with an altered gut microbiome.

The interest on the host-microbiota interactions and its health implications has been growing during the last years as demonstrated by several metagenomic studies of the human gut^6^,^10^,^50^. Detailed mechanistic studies and generation of new hypothesis through genome-scale modeling is a necessary complement to the statistical correlations revealed by metagenomics studies. Our study demonstrated that the metabolic differences in the gut microbiota can be identified through genome-scale metabolic modeling of bacteria in the gut ecosystem and this may allow for facilitating rational design of prebiotics and probiotics and for development efficient treatment strategies for different disorders.

## Methods

### Reconstruction of GEMs for *B. thetaiotaomicron*, *E. rectale* and *M. smithii*

The *iEre400, iBth1201* and *iMsi385* were reconstructed based on *Eubacterium rectale* *ATCC 33656*^33^, *Bacteroides thetaiotaomicron VPI-5482*^51^ and *Methanobrevibacter smithii ATCC 35061*^31^, respectively. The template models *iMH551*^37^, *iAF1260*^36^ and *iVS941*^38^ were used for reconstruction of *E. rectale*, *B. thetaiotaomicron* and *M. smithii*. The GEMs are available at the Human Metabolic Atlas (www.metabolicatlas.com) in SBML format.

By default the lower and upper bounds for reactions were set to ±1000 mmol/gDW^−1^ h^−1^, unless the reaction is irreversible. Following the RAVEN Toolbox for reconstruction the three template models were standardized based on databases with respect to metabolite names. The draft models were generated based on bi-directional BLASTp with respect to template models proteins and their orthologues in each interested species. The proteins were considered as orthologues with the settings of E-value < 10e − 30, sequence coverage > 50%, identity > 40% and alignment > 200 aminoacids. This gene mapping was controlled by identifying metabolic functions that were present in template models and not in draft models. Then the gaps were filled based on reference sequences database and literature. In addition a KEGG models for each species were generated by using RAVEN Toolbox and applied to fill the gaps in the draft models. The non-growth associated maintenance ATP were set based on available experiment data of the interested species or template model. The core biomass reactions adapted from the template models with respect to available information on biomass composition of the studied organisms.

### Simulations of α and β problems

The available experimental data are given in concentrations per unit of cecal content and no information is available about the production rate of cecal content per unit of time. By dividing all the metabolic fluxes by the specific production rate of cecal content (which is equivalent to the dilution rate of a chemostat), we can formulate the problem in such a way that the output fluxes will have the units of concentration that are available experimentally (Figures 2.D & 3.D).

In the paper of Mahowald et al, the levels of colonization of the mice have been measured and reported as genome equivalents/gram weight cecal contents. This has been done for both mono and co-colonization. We extracted these data and converted them to gr D.w./gram weight cecal contents by considering the value of total dry weight per cell in *E. coli* which is 2.8e − 13 gr. By considering the genome equivalent as a cell and multiplying the level of colonization with *E. coli* dry weight it is possible to calculate the concentrations per unit of cecal content. In the α-problem the objective function is minimizing the substrate uptake by constraining the biomass of single or community of species. In the β-problem the objective is maximizing the individual or community of species by constraining the substrate uptake. The optimization α & β problem solved therefore takes the following form: 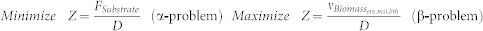

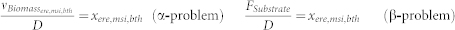

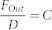

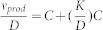

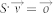



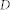
: Dilution rate


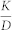
: Ratio of SCFAs absorption

*Prod*: Butyrate, Acetate and Propionate

### Reporter metabolites determination

Gene expression data generated by Mahowald et al^33^. was downloaded from Gene Expression Omnibus with the accession number GSE14737. The data Cel-files were processed using R and Bioconductor. Data normalization was done by using Probe Logarithmic Intensity Error (PLIER) estimation. Differentially expressed genes were identified using the moderated t-statistic for pair-wise comparison. P-values were corrected for multiple testing by the method developed by Benjamini and Hochberg. The reporter metabolite algorithm was applied using an in-house developed software platform PIANO^52^. The algorithm makes use of p-values and the gene-metabolite associations to identify metabolites associated with transcriptionally changed genes.

### Random sampling and transcriptional regulation identification

We have implemented a previously developed algorithm^35^. In order to avoid loops in the solutions, the upper and lower bound were set to Inf and –Inf respectively instead of 1000 and -1000, which is a common practice. To set the problem for running random sampling algorithm the exchange fluxes were taken from solving the α-problem. In the case of *B. thetaiotaomicron*, the exchange fluxes from two solved α-problem where *B. thetaiotaomicron* is single and is together with *E. rectale* were used as input fluxes to the random sampling algorithm. The same procedure for the case of *E. rectale* was done.

## Author Contributions

S.S. and J.N. designed the study. S.S. reconstructed the models for ere and msi, performed modeling and transcriptome analysis. F.K. reconstructed the model for bth and assisted with analysis. S.B. assisted with metabolic modeling. I.N. and A.M. assisted with transcriptome data analysis. J.N. conceived the project. S.S. and J.N. wrote the manuscript and all authors read and approved the final manuscript.

## Supplementary Material

Supplementary Information Supplementary information

Supplementary Information Supplementary Dataset 1

Supplementary Information Supplementary Dataset 2

Supplementary Information Supplementary Dataset 3

Supplementary Information Supplementary Dataset 4

Supplementary Information Supplementary Dataset 5

Supplementary Information Supplementary Dataset 6

Supplementary Information Supplementary Dataset 7
