# Supplementary material for: Understanding the interactions between bacteria in the human gut through metabolic modeling
Source: Sci Rep. 2013 Aug 28;3:2532. doi: 10.1038/srep02532 (PMC3755282; doi:10.1038/srep02532)
Supplement: Supplementary Information [file srep02532-s1.pdf]

Understanding the interactions between bacteria in the human gut through metabolic modeling

Running title: Modeling of human gut microbiome

Saeed Shoaie, Fredrik Karlsson, Adil Mardinoglu, Intawat Nookaew, Sergio Bordel and Jens Nielsen*

Department of Chemical and Biological Engineering, Chalmers University of Technology, Kemivägen 10, SE412 96 Gothenburg, Sweden

Email: saeeds@chalmers.se, frekar@chalmers.se, adilm@chalmers.se, intawat@chalmers.se, velasco@chalmers.se, nielsenj@chalmers.se

* Corresponding author

E-mail: nielsenj@chalmers.se
Tel: +46 31 772 3804
Fax: +46 31 772 3801

Supplementary figure, table and datasets legends:

Table S1: The characteristics of models.

Figure S1: Validations of the *iBth1201* based on available *in-vitro* chemostat data.

Figure S2: Robustness analysis for α-problem, testing glucan prediction for different biomass values.

Figure S3: Robustness analysis for α-problem, testing SCFAs prediction for different biomass values.

Figure S4: Robustness analysis for β-problem, testing biomass prediction for different glucan values.

Figure S5: Robustness analysis for β-problem, testing SCFAs prediction for different glucan values.

Figure S6: Complete reporter subnetworks, when *E. rectale* was responded to *B. theta*.

Figure S7: Complete reporter subnetworks, when *B. theta* was responded to *E. rectale*.

Figure S8: Sensitivity analysis of *E. rectale*: Identifying optimal production of biomass.

Figure S9: Sensitivity analysis of *E. rectale*: Identifying optimal production of butyrate.

Figure S10: Sensitivity analysis of *E. rectale* and *B. theta* : Identifying optimal production of butyrate.

Supplementary Dataset 1: Lists of applied tasks for validation of GEMs.

Supplementary Dataset 2: Integrative analysis of transcriptomic data for gut microbial communities, reporter metabolites (respond of *E. rectale* to *B. theta*).

Supplementary Dataset 3: Integrative analysis of transcriptomic data for gut microbial communities, reporter metabolites (respond of *B. theta* to *E. rectale*).

Supplementary Dataset 4: Integrative analysis of transcriptomic data for gut microbial communities, reporter subnetworks (respond of *E. rectale* to *B. theta*).

Supplementary Dataset 5: Integrative analysis of transcriptomic data for gut microbial communities, reporter subnetworks (respond of *B. theta* to *E. rectale*).

Supplementary Dataset 6: All the probability scores for each metabolic flux for transcriptional regulation, when *E. rectale* was responded to *B. theta*.

Supplementary Dataset 7: By using these transcriptome data, the random sampling algorithm and GEMs for ere and B. thetaiotaomicron, the transcriptional regulation for when the B. thetaiotaomicron was adapted to ere were identified.

Table S1 Characteristics of three models			
**Models**	iEre400	iBth1187	iMsi385
**Genes**	400	1187	385
**Reactions**	434	1386	490
**Metabolites**	381	1352	379
**Compartments**	2	3	2
**Gene coverage**	11%	24%	21%

Table S1: The characteristics of models.

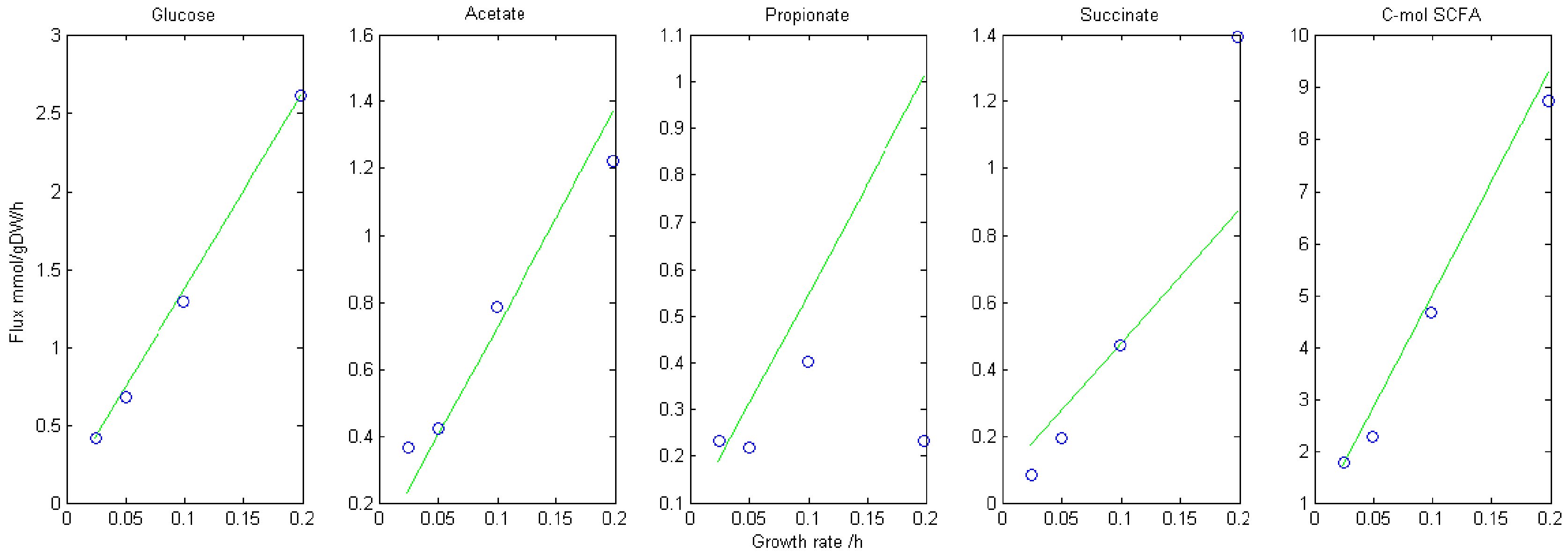

Figure S1: Validation of the model predictions were done based on available in-vitro chemostat data for iBth802 (Salyers et al., 1982).

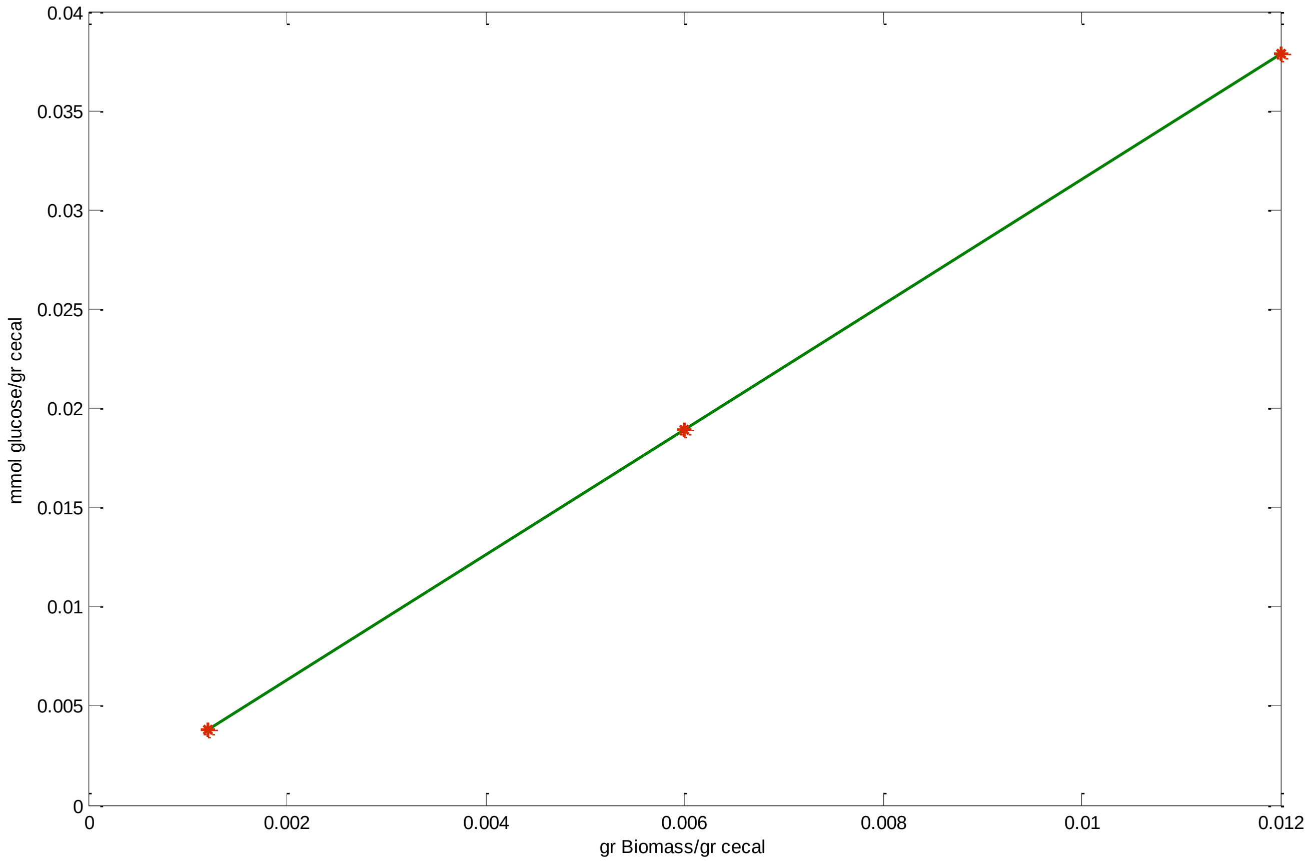

Figure S2: Robustness analysis for α-problem, testing glucan prediction for different biomass values.

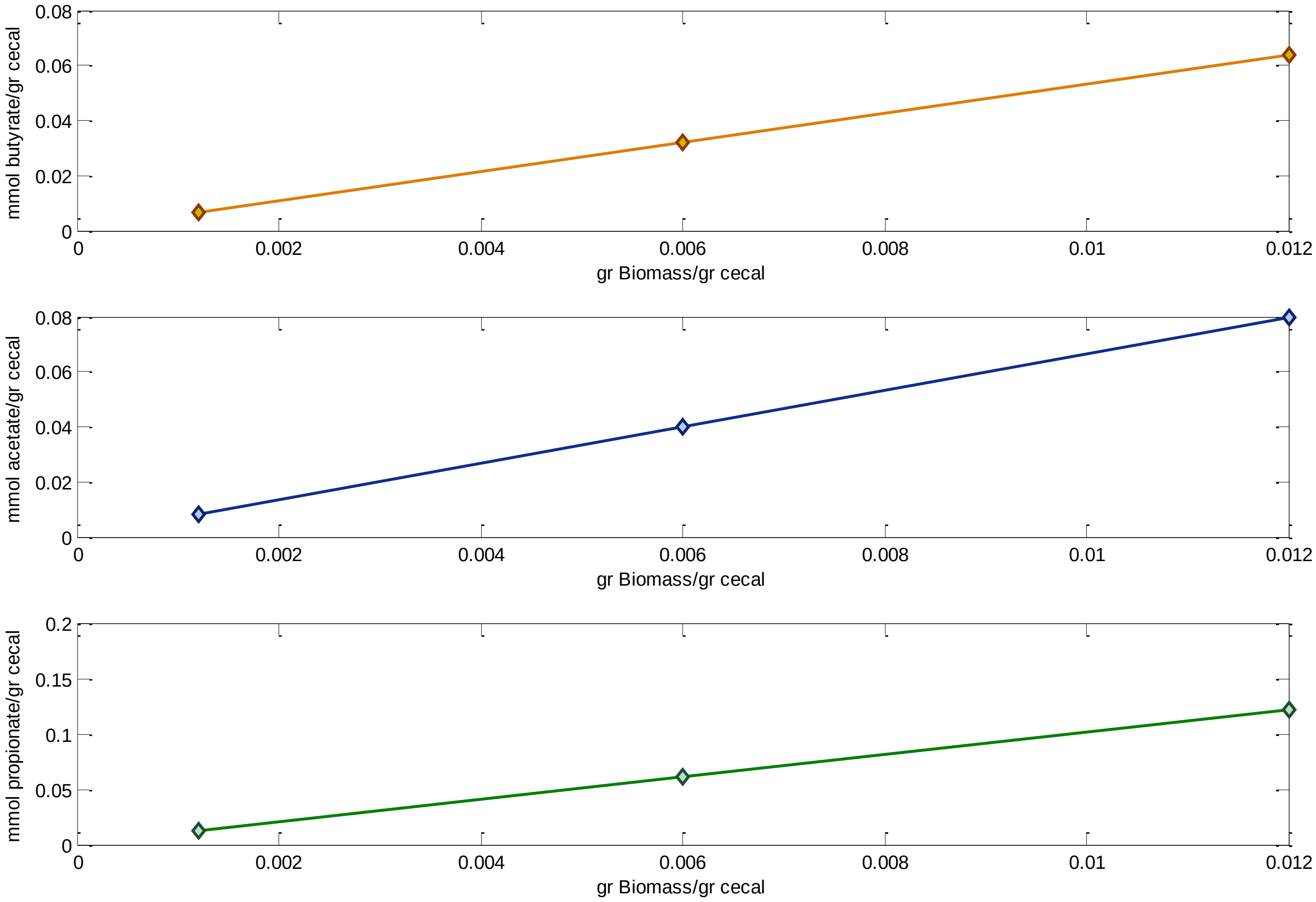

Figure S3: Robustness analysis for α-problem, testing SCFAs prediction for different biomass values.

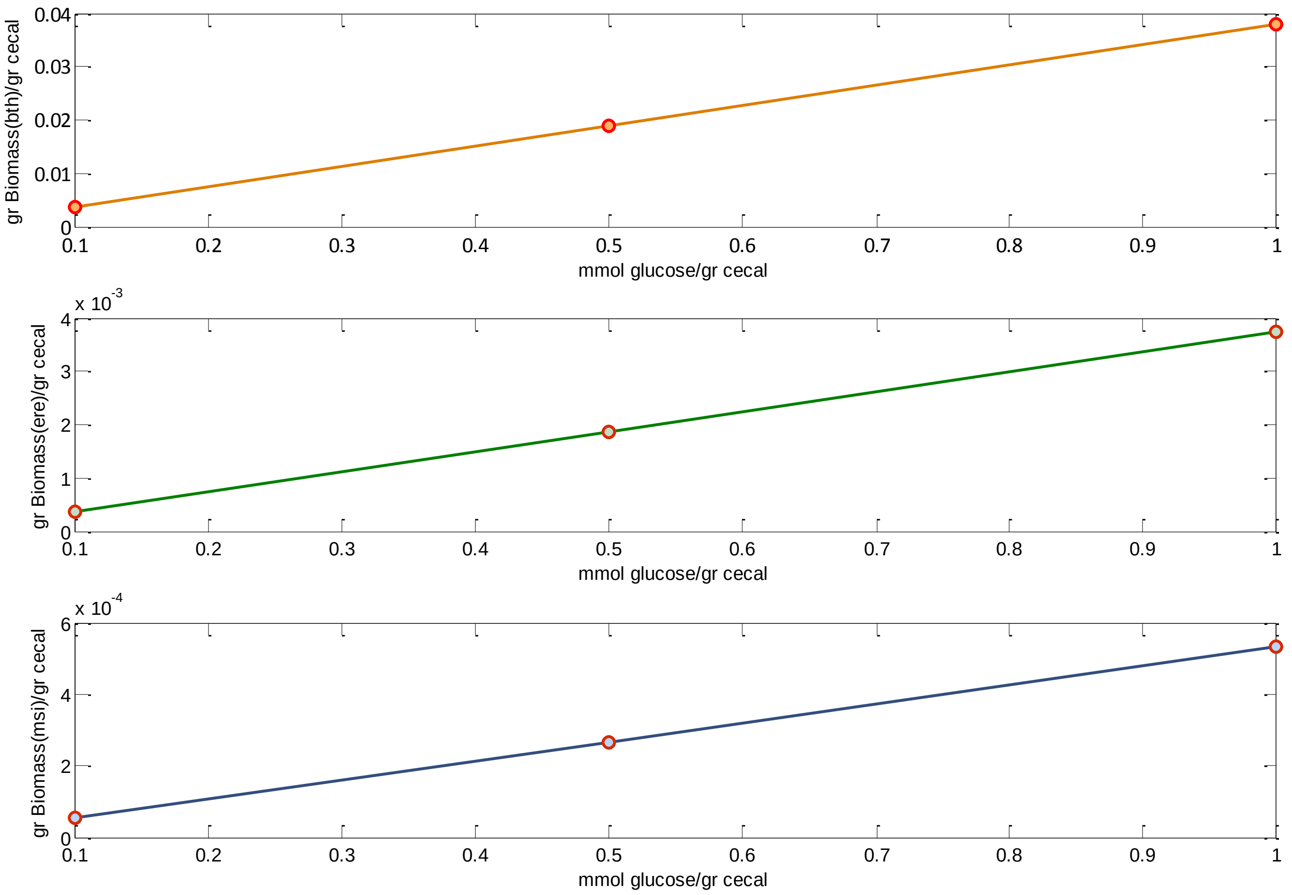

Figure S4: Robustness analysis for β-problem, testing biomass prediction for different glucan values.

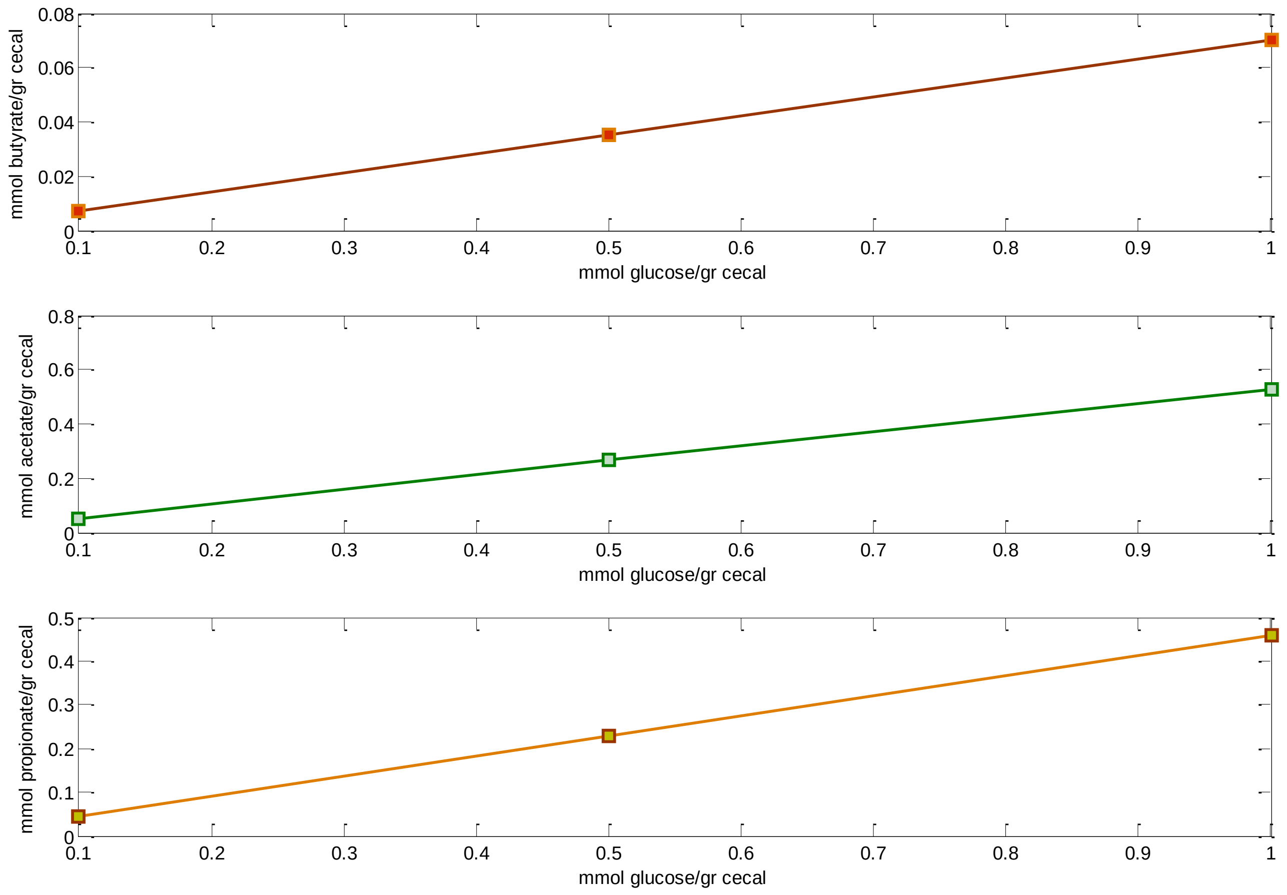

Figure S5: Robustness analysis for β-problem, testing SCFAs prediction for different glucan values.

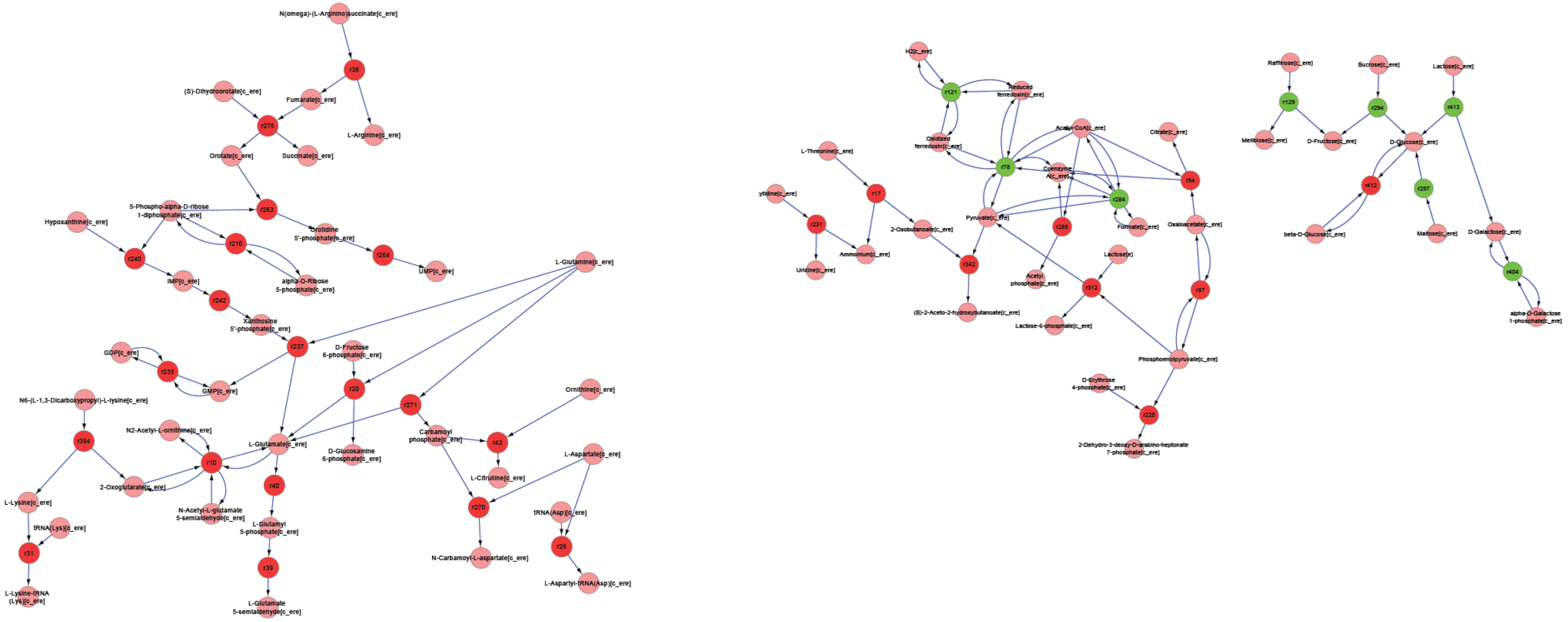

Figure S6: Complete reporter subnetworks, when E. rectale was responded to B. theta.

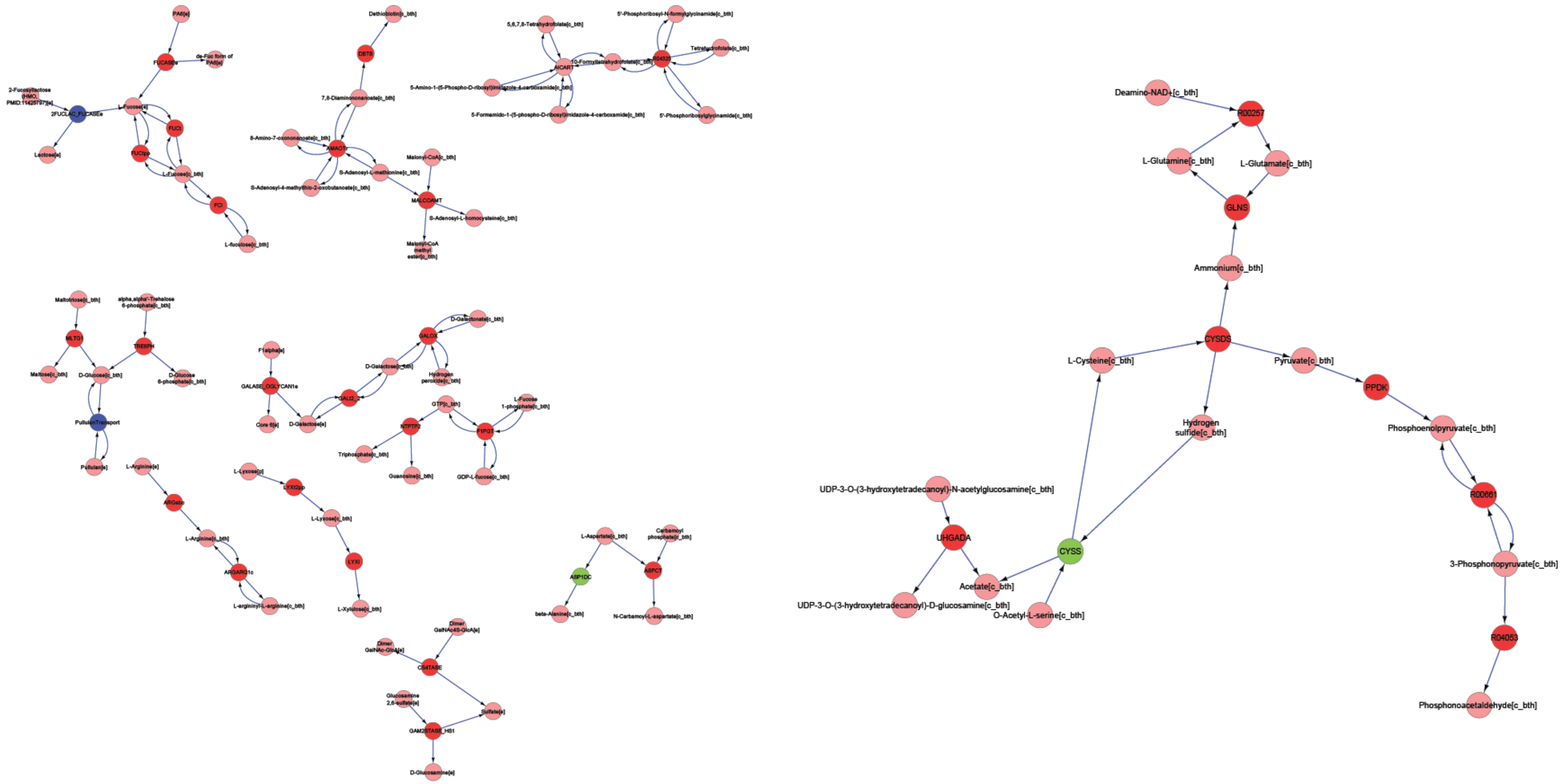

Figure S7: Complete reporter subnetworks, when *B. theta* was responded to *E. rectale*.

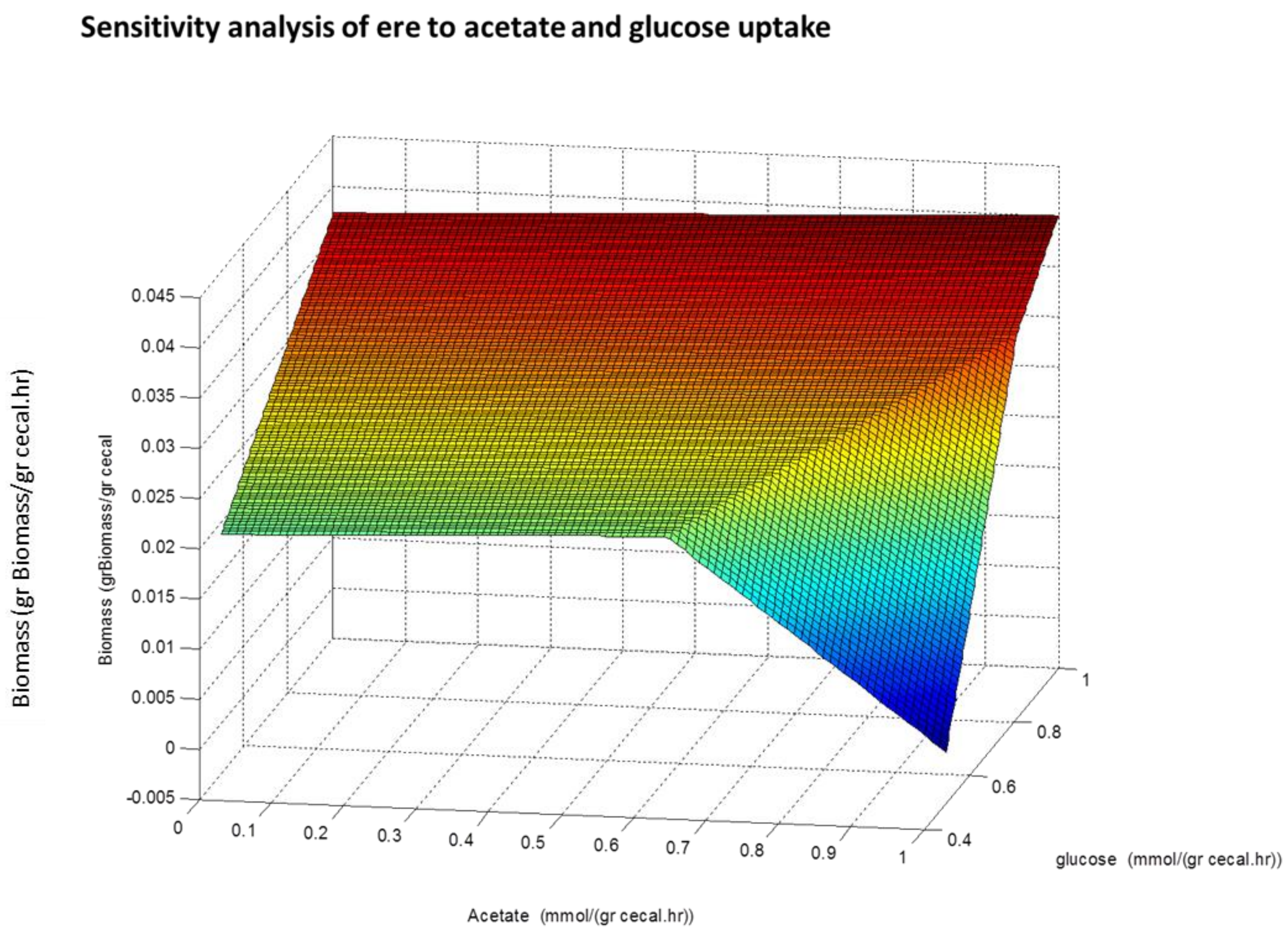

Figure S8 Glucose uptake rate was varied between 0 to 1 mmol/grCecal, and the value for acetate was varied from 0 to 1 mmol/grCecal. The simulation were done based on maximizing the biomass as objective function.

Sensitivity analysis of ere to acetate and glucose uptake

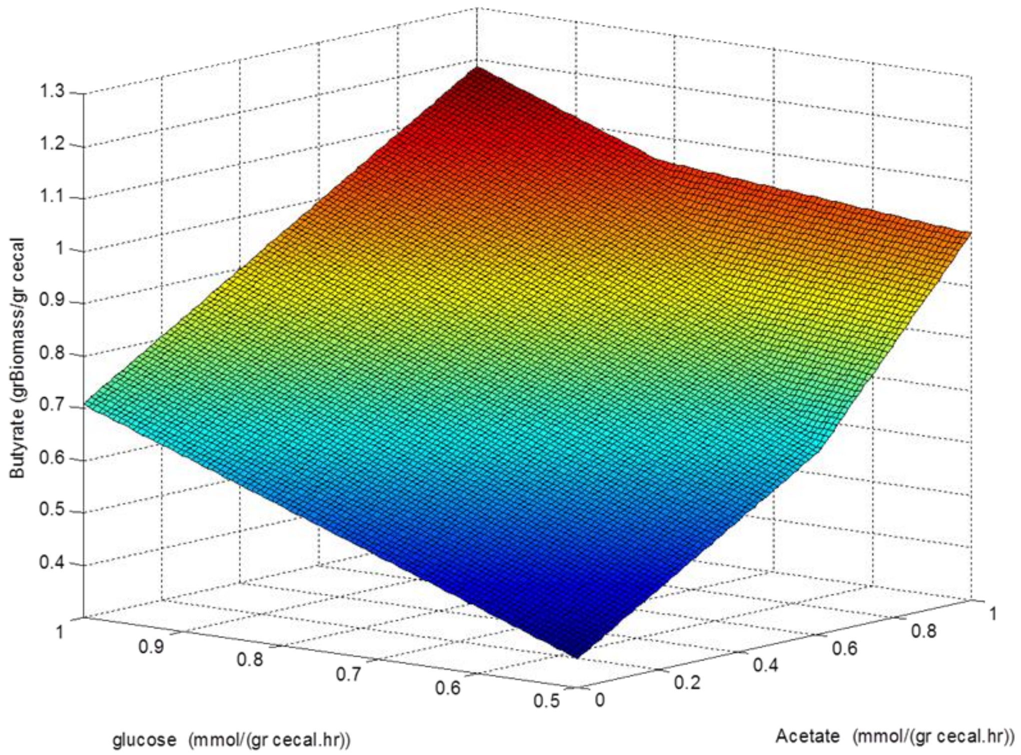

Figure S9 Glucose uptake rate was varied between 0 to 1 mmol/grCecal, and the value for acetate was varied from 0 to 1 mmol/grCecal. The simulation was done for maximizing the biomass as objective function.

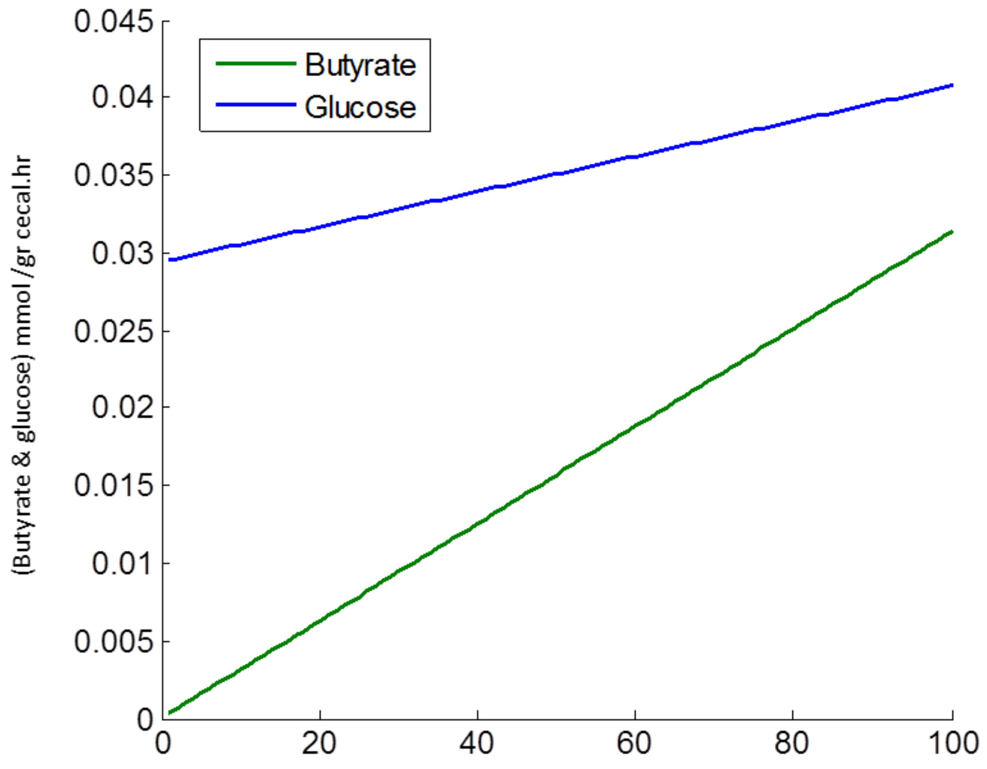

Figure S10 Sensitivity analysis of Ere and Bth; abundances of Bth and Ere were varied to check the butyrate production. The abundance of Ere in percentage (0 to 100%), where x=0 means the abundance of Ere is zero and Bth is 100% of 0.0012 gr biomass/g Cecal and vice versa.
